# Supplementary figures and images for: Cnidarian hair cell development illuminates an ancient role for the class IV POU transcription factor in defining mechanoreceptor identity
Source: eLife. 2021 Dec 23;10:e74336. doi: 10.7554/eLife.74336 (PMC8846589; doi:10.7554/eLife.74336)

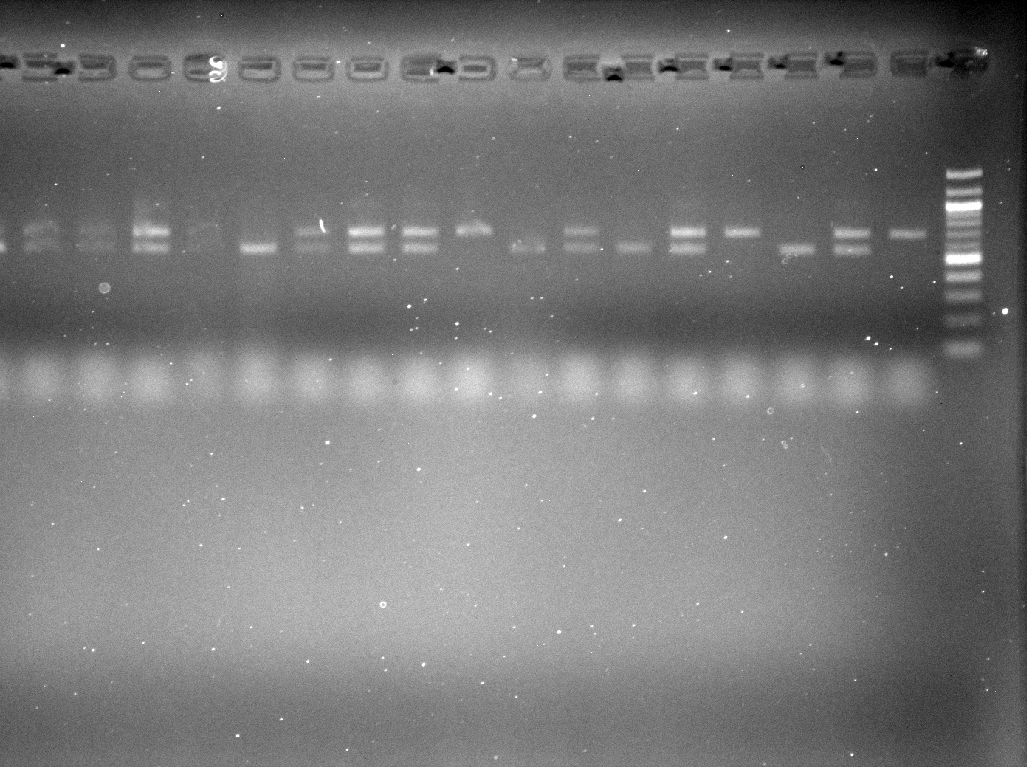

Supplement: Figure 3—source data 1. [file elife-74336-fig3-data1.zip › Source data for Figure 3C.tiff]

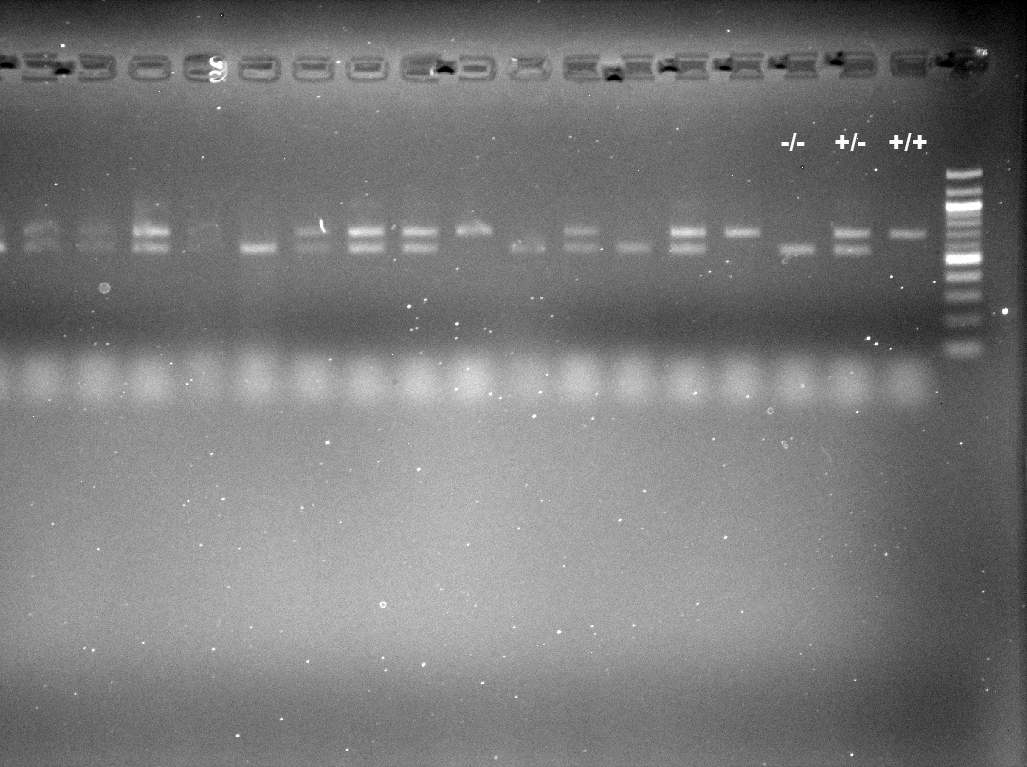

Supplement: Figure 3—source data 1. [file elife-74336-fig3-data1.zip › Source data for Figure 3C labelled.tiff]

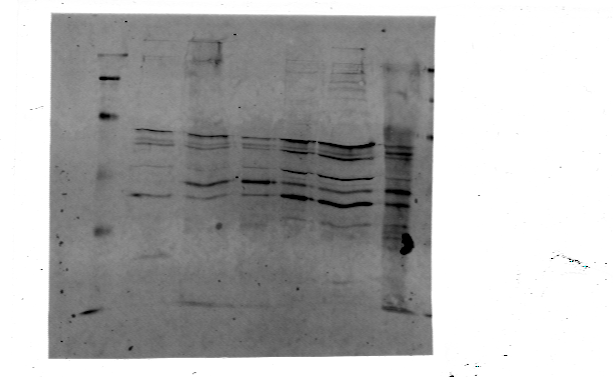

Supplement: Figure 3—source data 2. [file elife-74336-fig3-data2.zip › Source data for Figure 3D anti-POU-IV.tiff]

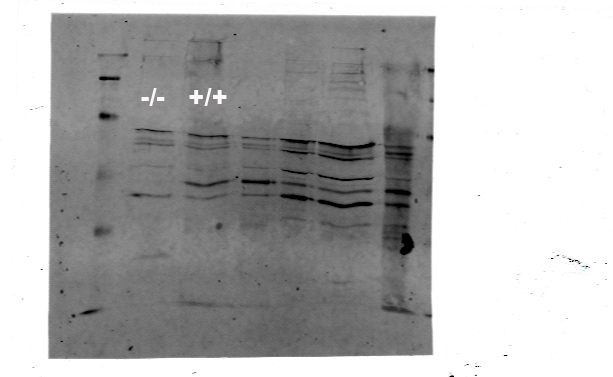

Supplement: Figure 3—source data 2. [file elife-74336-fig3-data2.zip › Source data for Figure 3D anti-POU-IV labelled.tif]

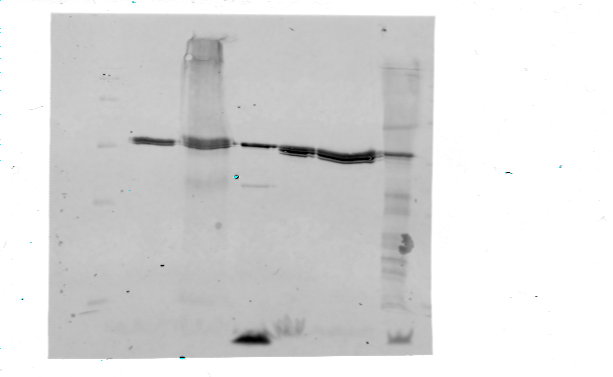

Supplement: Figure 3—source data 3. [file elife-74336-fig3-data3.zip › Source data for Figure 3D anti-tubulin.tiff]

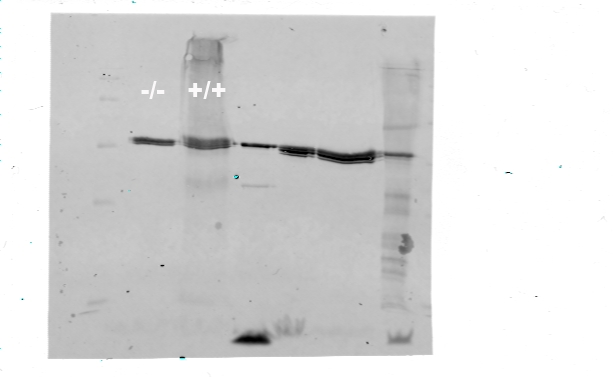

Supplement: Figure 3—source data 3. [file elife-74336-fig3-data3.zip › Source data for Figure 3D anti-tubulin labelled.tif]

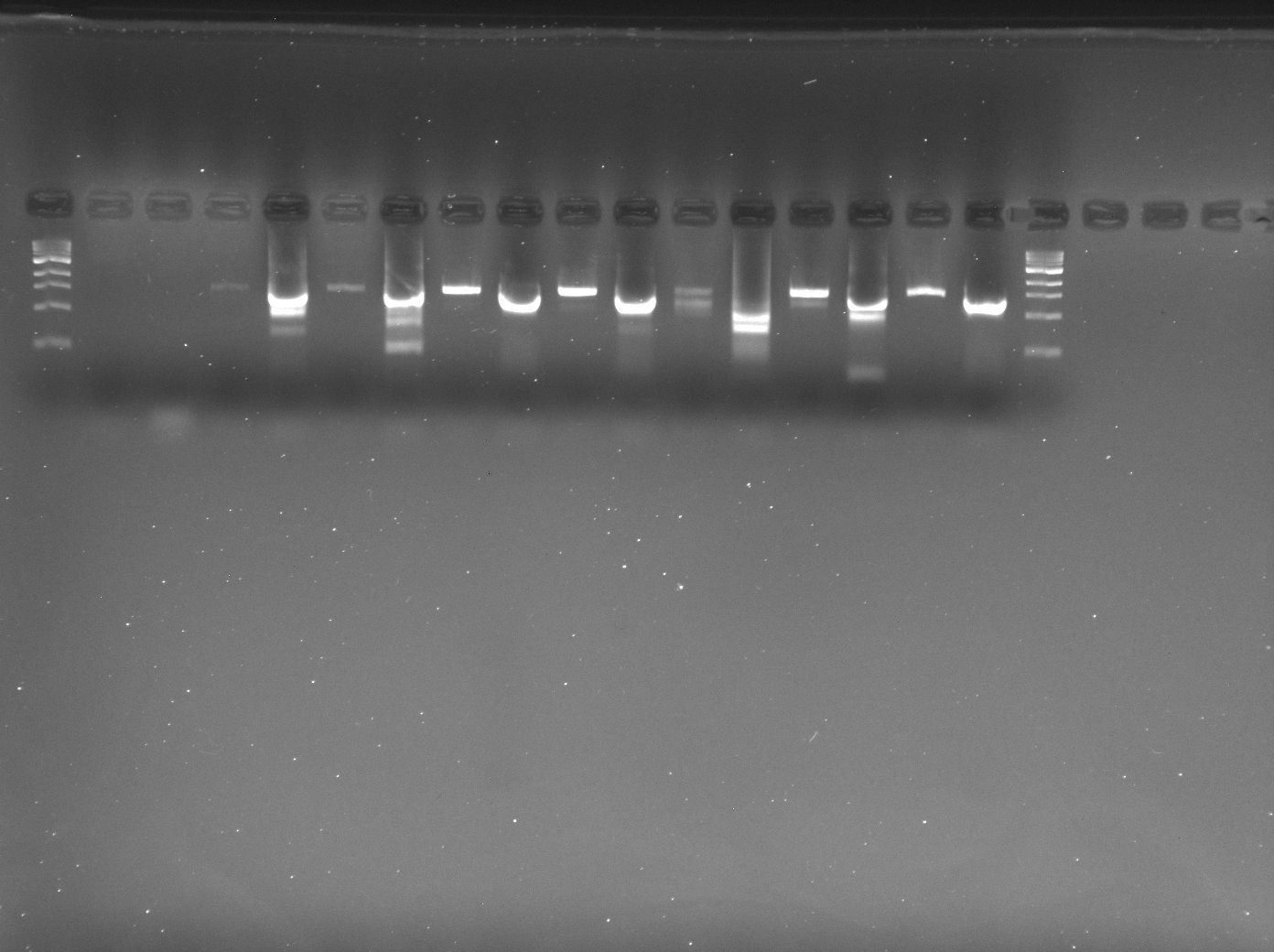

Supplement: Figure 3—figure supplement 1—source data 1. [file elife-74336-fig3-figsupp1-data1.zip › pou-iv crispr F0 genotyping.jpeg]

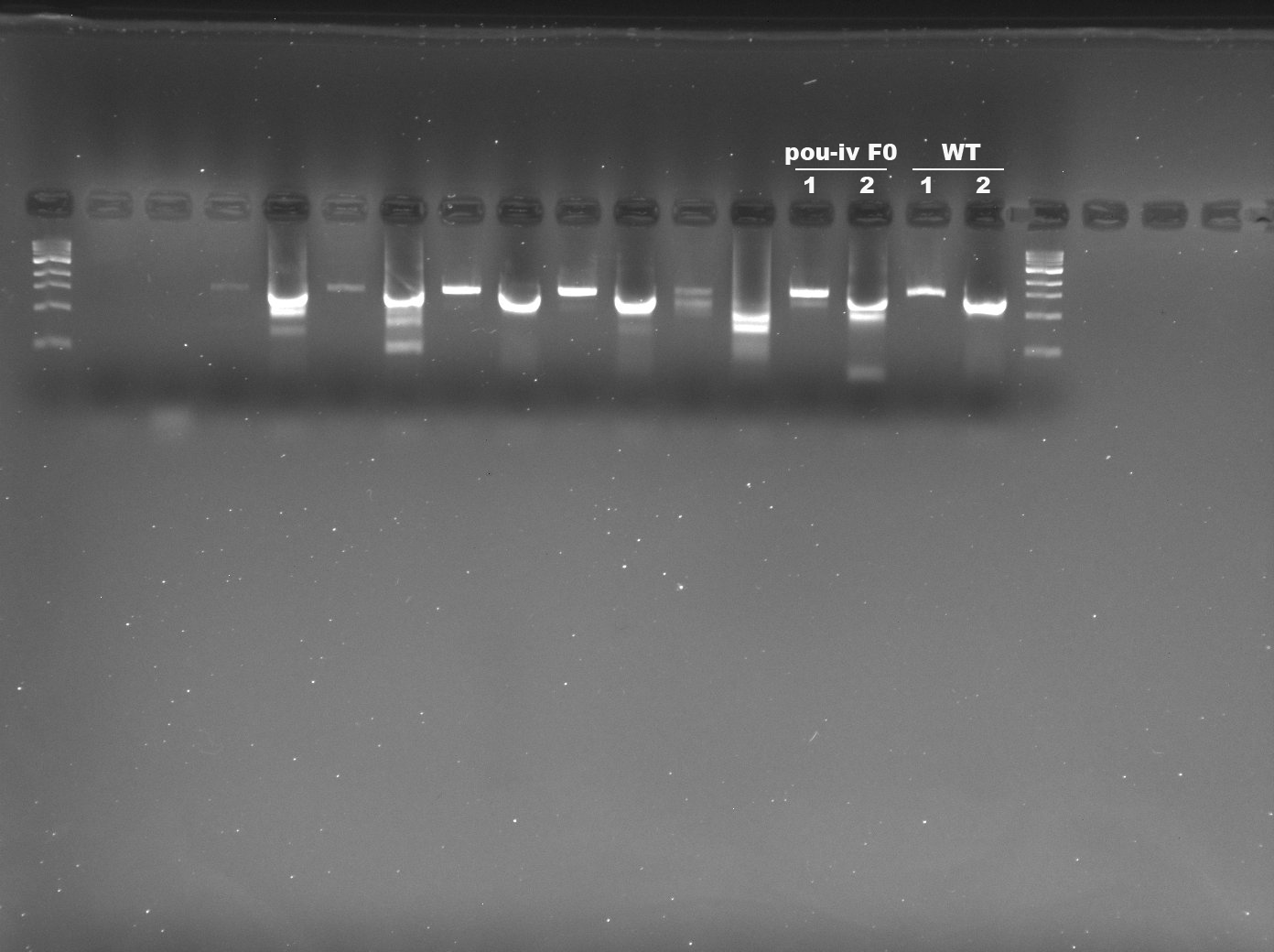

Supplement: Figure 3—figure supplement 1—source data 1. [file elife-74336-fig3-figsupp1-data1.zip › pou-iv crispr F0 genotyping with labels.tif]

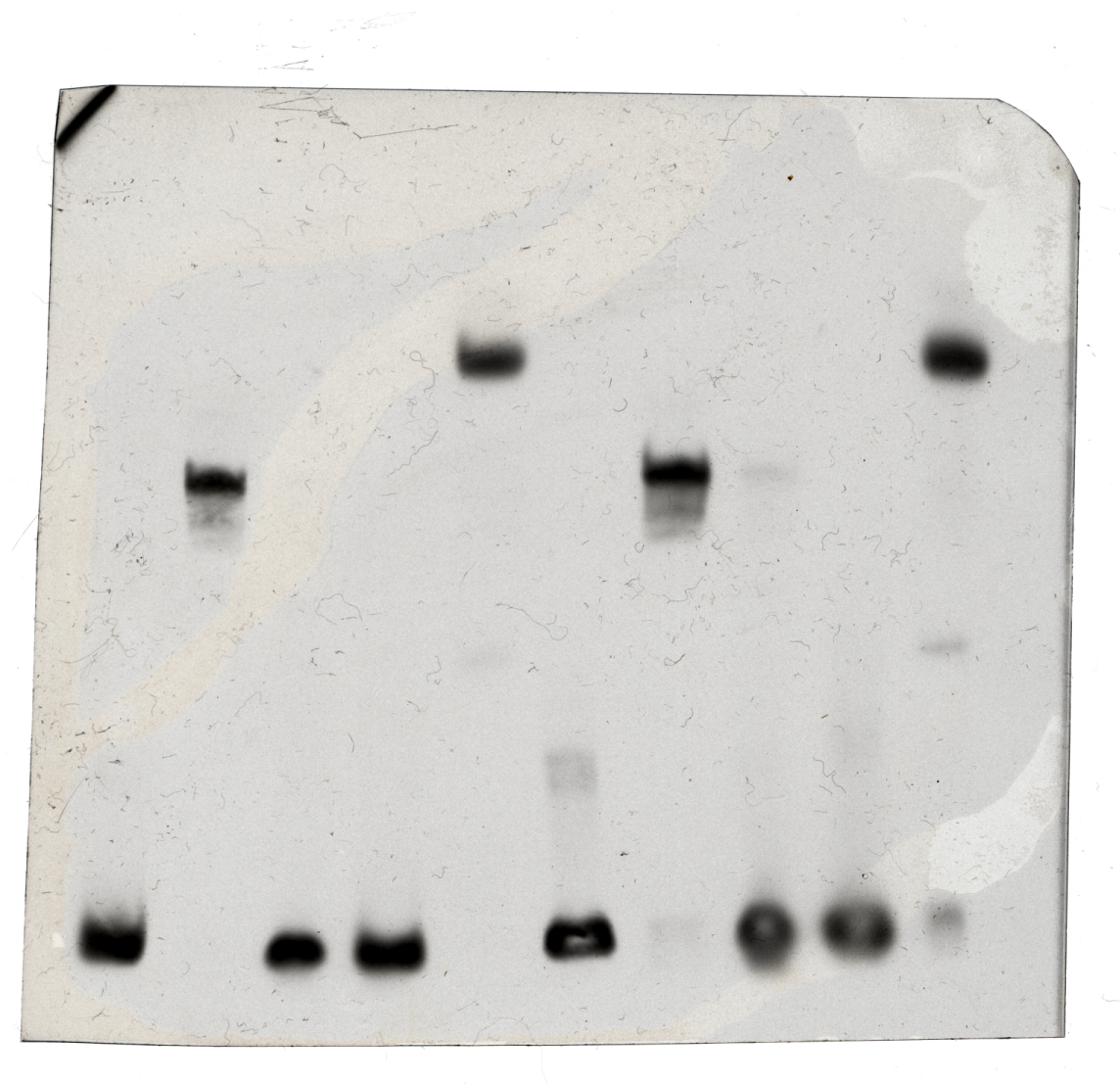

Supplement: Figure 7—source data 2. [file elife-74336-fig7-data2.zip › Figure 7 - source data 2.tiff]

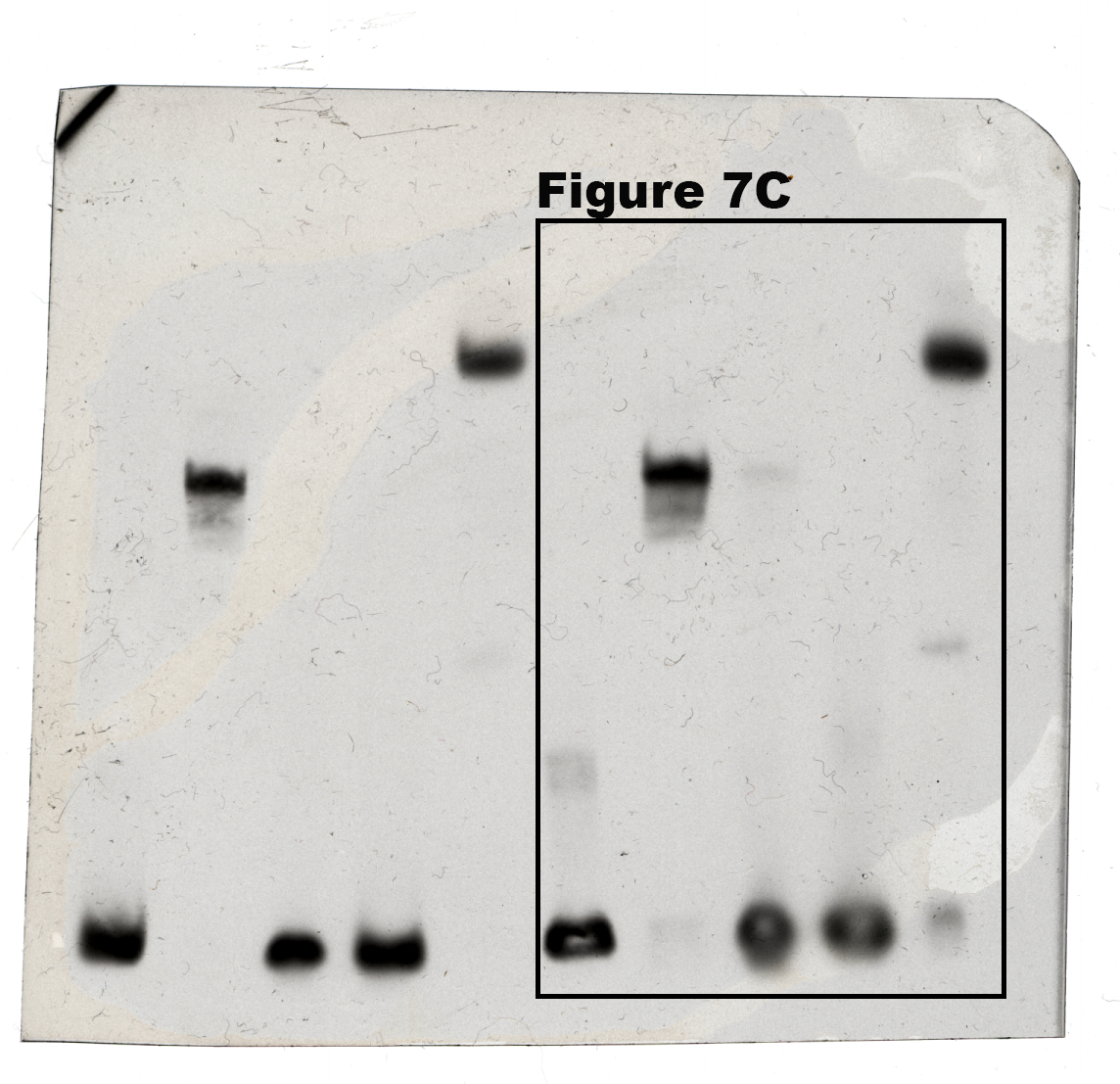

Supplement: Figure 7—source data 2. [file elife-74336-fig7-data2.zip › Figure 7 - source data 2 labelled.tif]
