# Supplementary material for: Cnidarian hair cell development illuminates an ancient role for the class IV POU transcription factor in defining mechanoreceptor identity
Source: eLife. 2021 Dec 23;10:e74336. doi: 10.7554/eLife.74336 (PMC8846589; doi:10.7554/eLife.74336)
Supplement: Figure 8—figure supplement 1—source data 1. [file elife-74336-fig8-figsupp1-data1.docx]

AGGGACCATCTTGGCAAGAGCAGCTCACATGAAAGACGTAGGTCGTTTTACCGAAGAAAAATAGACAAGGCAACAACTAAACTGCCGCAAAACACAACCAAACAATGACGACCAAAAGATATCTGTGTGTTCTTCTCTGTCTGACAAGTTATTTGACTTATTCTTCTGGTATGATGACACAAGTTCGTAAAGAAGAATACCTTGGGTGCTTCACGGAATCCGAAGAGTCCCGGGTTTTTTCCTCCGGTCCGGGGGACTATGACCCTCATGATATAAGTCCAATACGATGTTTGGAGCAATGTGGTATCAAGTACAAGTACGCAGCACTACAGGATGGGAGATTGTGTTTGTGCTCGAATACTCTACCAGGCACTCCAAAACTGGACGACTCAGAGTGCAACACACCTTGTCCAGGTTCTAGTAAGTGGCCGCCTTCCGAGCATTACCTCAAGTGCGGTGGACCCCTGAAGAACAGCGTATATAACGCCGGAGAGCGCATTTTGGGGTTTACACTACAGAAGATAGAATCGTTGAATATTTTAGAGCCGGTCAATATCCATGGTGGAATTACCAACGGGATTAATGTGAGTTATGTCTTTGACTTGGGCGATGGAACGCTGGTAACAAAGCCTTCGAGCGAACCGAAAGCACGCCATATATATGACAAACCTGGGTCGTACGTGGTGACTGCTACAGCTAGTAATATCATCTCTGGTGAGGTGGTGGCCTCGGAGGTGTATAATGTGGACGATCCTAGGAATAATATCCGGTTAACGTGTCCAAGGGCAGCTGAAGTTGGACAAATCGTAGAATGTAATGGAACGATGGACCGCGGATCGCGTGTGAACTCCACGTTTGTGTTTTCAGATGGTCGGACCGACCGAATGTCTATCAGCTCCAGGTATTATAGTGCTGGTACGATTGTTCCTCGAGGGAACGACTCGTCCGTCATCCCCGTGCTCAATACCCCAGGGACTATCCTAATCCCGGCGTACGAGTTCCAACACGATGGTCAAGTGACGCACTGGGACTTCGAGATCGTCGAGAAAGGAACAATCAAACTGATGATACTCCGCCCTGAGTGTTCCGCTGGGGAAGAATATTGTACATCTACTCGCTCCTGCAAGATCTCATCCTCCTCCTGCCTCCCCCTTAAACAGAAAAAGTGTTCCTCCGATGAAATGTTCTGTATGATTCAGAAGCGCTGCGTCAGTAACGCTTATACCACAACCCAAGACGCCAACAACAACCCTGTCAAGGTCTACACCAGCAGCAGCACCTGCCCAATACAAGCCCCGTACCAATGGTCCGAGCCGAGAGCCGACTACAGAATCTTGTTCGTTCAGGAAATAAGCCTGGAAACTATTGGGCATCACATCTCGGCAATCCCACTGGCGCAGCAGCCGTTTGTTAAAGAAGGTGACATACTGGGCTGGTTGCCCGTGACTGGTTACCTAGCGTACAAGTCGGTAGCTGATCACGAAGGTGCGTCTTTCGAGTATAGCTCCGGTGTGTCAGCTGTGAATGACAAACTTCTCCGCAGCGGTTCCACAACACTTCACCAAAAGCATTTCGTATTCGCCGCACATTACGCGCATGTGGCCAAGTTTGTGGTCCGAAATCGTTTCGGGACTCCAGGTTTGAAATCTCTTACCTCGAACATTACCGAACCATTGTACTTATACATCGATTATCCTATTCGTAATGTCACATTCGAGGCAAGCAAGTTCGCGAATACAAACGACTCAGTTGAATTCCTGGTTCCTGAACATCCCGGCACAAATACTACGTACTTCTGGGACTTCGGCAACGGTGAATCTTTGTATACCCACTTGCCGTCAATCTCTTACGCCTTTCCAACTGAGGGAGTTTTTTACGTGAGCTTGCGAGCTGAAAACTCCATTAGCCATACCGTCCTCACATTTCCAATCTCTATCTTTGATCCTATCCTCGAGTTCGAATATAAATCCCCAATTAAAGCAAACGCGCTAGGCACTGAGACGCTAATAGAATGGAAGACATCTCGGGGTACCAATATTACATTTGTTGTTGACTTTGGGGATGCCACACCTAGGTATTCTGCGGTCACTACGTTATCCGGAGGTAGGGCTGTTGATACTAGGCATACATACTCCGCTGTTGGGAACTACACGGTCACTGTGTACGCGTTCAACAGAGTGGGGCCGAACATCACTATAGTGTCCTATGCCGTTGTGGAAGTCCCACTAGAAGGTCTAGAGTTCAGCGTGCCCAACCCTCATATCACTAAGAATATCTACCTGGCTGCCGGGGACACGATGACTGTCTCGAGGCACTACCAGAAAGGCACTAATATCAAGTGCTCTTGTGACTTTCGAGATGGAACCCCACCTGTACTGACGACAAGCCAAGACATGAGTCACACCTACACAAACGCGGGGACATACCATGTGGAAATCACCTGTTTCAATGACGTGAACTCCATAACCAAACCACTGAATGGAACTGTTGTAGTCCAGGAACTACAGGCAATTACTGGCTTGACAGTCCTTACTTCGGCTACAAAGTTCGGAACTCGCTCGGAGCTTTTGCTAGAAATGGCTACAGGGTCAGTATTTGTTTGTGAGTGGGACTTCGGCGATGGGAACAAAACCAGCACTGACTTCTCGTTTATGGGGCAAACAATGTATTATACTTATGTGGCAGTCGACACTTACAACGTGGCAGTCACGTGTACAAACAGAGTAGGATCCGTGACGGCAAGGGCAGTAGCGCCAGTCGATATACCAATTGACGGCGTGATAATCAGCAACAACAAGCGTTACATCAAGGTCGGCGAACCAGTGCGCCTCGACGTCACGGTACAAAAAGGAACGAGAATGCTTTACACGGTCAGCTATGGTGATGCTTCGACGGGATCCTTGTCACGTGACGCAGCGAAGGCACCGAGTTTGGCCGATCACGAGAGTTTTACGCATGCGTACGCCACCGATGGTTCTTACACAGTCAAGGTTAATGTGTCAAACTCGTATGGCTGGAAGGAGGAGACTTTGGGTGAAACAATTATGGCGCAGTACCCAGTAGAGGGAATCATCCTGAGGTCAAATTCCCCGGTACGGCTATCATCAGGAAATGTGACTTACTTCATCAGCGTCCTTGAAGGCGCGAACCCCCCTACAGGGGCTTATGCTGTGTGGTCTTTTGGTGACAACAGCCCAGTGACAACACCCGAACCCATTTACGACTTGCGACAGAAAACGTATATGCGCTCCCATCGATTCATGATCAATAACACATTCACGACAACGGTGAACATTAGTAATCAAGTCAGTCATGAGGTGTTGGCCATCGACGTTCGAATCCAGATGCTTGTTGGGGTGATTATCACTCCGCTACTGGTGACTAACGCGACGTTATTCACCATTACTAATGGTTACGGACCGGAGATGAACTATTTTGAGGTTAATAAGCTGATTGCGTTCACCTCTTCCTCCCAACTTGGTGATCGAACCTGGGCATGGGAGTTTGGCGATGGAGCATCCACAAATGTGTCGAGTATCCCTACTTCTACCCATACGTTTAATACCTCAGGGACCTACGCCGTGCGAGTAGTCGTCAATAACTTTCTTGACGTGCTGGAGGCGGAGAAGACTGTCTTTATACAGGACCCGGTAGGGAATGTCACCCTGAGCTCTCAGCTACCAACGTACTACCGGGAGCCAACAGTCTTCAACTTCCAGGTCACTTCCCGCGGCTCACAGTCGTGCTTGAAACTAAGCCTCGGAGACAATAACGGCGCCATTTTCGGTCAAAGGCATTGCAGGCCTAGCGTGATGGTGGCTAACGTGACATTTATCCCCGTCCCTGAAAACCAGACTTCGTTCAACTACAGCTACATGTATATTTATCGGGGGAACTACTCGGTGGAATTGACCTTGTGGAATTTTGTGTCATCTCAGTCTGTAGTTTGGCCTATAGAGATTGCAGATTTGCCTTGCGACTATCCCATTGTCAGAATCGACAGCGAGGGAACCAAGACAAGCCCTCGTAAGGTGAAGAAGTCAGAGCCGCTCGTACTCCCTGCTGATGTCAGGTACAAATGTCCTGTCGGCAAGAGGATCATCTTCTCGTGGAAGGCATATGAGGTAACCTTACTCAACCCAGACGACGAAAGCAGGCCATTCAATCTCCCTGTGAACGAAATAAAGACCTTTGACCTCCCTGCCCGTGATACTATCATGGATGCAGGGTCAATAAAGATCAAAGAGCGCACCTTTCCCTTCATTACCCTCAAGTTCACTCTCGAAGTCGGCTTTGTCGGGTCCGATAGAGACTTGACTCACTTCACGCATAGCCACAGCGTCTGGATAGAAGTAGAAAAGTCCTTGCTGTATGCTGTCATTAGAGGCGGCCAGCGTAAGTCAGTGGGTTACGAGATGGATATGCTTCTTGATGGCTCAGAGTCCAAGGACCCCGACAATCCGACCAACACAACCGGGATTGTCTACACGTGGTGGTGTCGTAGAGATGAAGAGAGCTTTCCATCAGCTTTCGACGCGCCTAACCCTACCGGGGGATGCTATGGAAACGGGAATTACCAACTCAACGGCTCGACCTCAGAAATCTCTGTCTACACAGGCGCTTTCCTTCAGAACGCCGTCTATGTGTTCAGAATCAAGGTGGTTAAGGAGGAGCGTGAGGCTCTGTTTGATCAGTACATCACCATACTGCCCGGACAACCTCCGACTATGAACCTCAAATGTAACTTCAACTGCCTGGCCAAGACCAACCCCATCGAGCGTCTGGTCATGGAGACCACTTGCCAGGACTGCAAGCCGACCGACATCCTGGGATATGAGTGGTCCCTCCACCGCCTGCTTCTCGGCAAAGACCCCGACCAGATTGACTCCTGGGAAACCATTAACCCTACAAGTTGGGCGGTCAACACCTCTACTGGGATCGACAAGGGCAACTTGGTCATCAACTCACATTTCCTTGAACCGAGTCGTAGCTACTTCCTTCGTCTGAATGCCTGGAAACCGGGAGGGTACCCAGGAGGTTTTGTCGAGCACAGGTTTACCGTAAATACCGCACCTACAAGTGGTTCCTGTAGTGTTGATCCTTTGGAAGGCTTTGCATTGGATACAACATTTCAAGTGAAGTGCGACGGATGGGTTGATCCTGACACTCCTCTTAAGTACCTCGTAGAGTTACGCAATGGCGCGGACATTGTGCCCATCTCGGATGGCTTTGAGCCGTACACATCAGCTGTGTTCCCTCTTGGCAAGGAAGAGAACAACTACACCCTCACGGTCAATGTAAAGGTCATGGATATGTTCTTCCTGGATGCAACAACCAAGTTCAGTGTCAGGGTAACAGAGCCAATCACAATCGATTATAACGAGGTAGGAGGTAGTGTTGCCTCGGCTGCGGGGTCCGGTAACGCCCAGGAGGCTACACAAGTCACGAATGCTGTCTGCTCTGTACTGAACGCCAAGGCCTGCAAGGAAGAAGACGATCCAAATGCTAAGGATGCCCGGGCTGACTTCCGTGGCGAGGTAGCCAAGTCGATGGCTACGTTGCCTGTTGACTCGTTCGACGGGGCGGCACAAAAGGGCGAGGCACTGAACGGGCTCACGGCAATGCCAGACGAGATCAAGGAAGATGCGCAGGAAGCGGTGACGGATGCGATGAACGAGATTGGAGACTTCCTGGCCAAAGACAATAGCGGTAGGAATCTGGACAACACTGCTAAGAGCCTTATTTCTGGAATTGGTAACATAGTTGGCGCGTCCAGTAACACGGCCAAGAAGGCGCTAAACTCCACCTGTGGAGACCCGTCTAAAAGCACAAACAACACCAAGAAAGCCTTAGACCTTGTCGAGATAGTTAGTAGCGCATGTATGAAACAACTGGTCGCAGGAGACAAGCCAAAAGCCATCAAGACCGATAACATTGACTTGGCAATAGGACGCAAGGATCTTAGTGATCTAGCGAACGACGACGAAGATGAAAGCGAGGGGGATACCGGAGGATTCAGCCTGCCAGATCCCGCTATGCTGTTCGGTGGAGCTAATGCATCAACTGAAGAGGGAGCAACAAGTGGTATCGGATCAACTATGACAGCCATGGGTGATAACCCTTTCCCTGGTGGCAGCGATGATCTCAACTCCAAGACCATCGGCTTATCACTCACAGATGGTAACGGTAACCCGCTAGACCTCGCGGGACAGACCCTCGAGATGTACGTCCCGCGGGATCTCAAGAAAAACCCGTTGAAACCGATGGAGCTGAACCACTTTGGCCCTAATGATCCTGTCATGAGAGTACACAAGTTTAACCGAACAACCAATCTTACCGCCATCGCTGTGGAGATCCAACCTTTCGACCCCCAGATCAAATTCCGCATCCATGTCCGATTCGAGACACGTCCCTCGGCCACCCACTTCCACTGGAATCACACCTTCCCGAGTCTAGAAGAAGCGGCCAAAATGAAGAGACGGCCACACCCCTTCACCTTCGTCATCAACCACGTGGTATTGCGTGACACGTTACTAAGTAGTAACGCAAGTGATAATGGGACCGTGTTCAACAGCACGATGGGCTCATACTTTTTGGGTATCAAGGCCATCAATAAGGACAGTTTGAGTAGCGCCAACACTAGCTACGCCATGAGGATCTACCTACCGGCGTGCAAGTCATTTGATGTAGACACTAACACTTGGACCACGAACGGATGTGTGGTCGGTAACAAAACCCGCGCGAATATCACCCACTGCGTGTGCCGACCAGGCGAAGACGAACCCGAGGACATAGACCCTACAGCTGTCCCCCCCGGCGCAGCTATAGGCAATGCAGCGTCCTCCACTGGAGACGACACCTCGTCAGGGGGGCCTGTTCGCGTCAGACGCTTCAAGCGGAAAAAGGTCTTCAAGCTTTCACTAGCGAGTAGTTTCTTTCCCGCGCCGAACCCCATCGACTTCGATAAAGTGTTCGCAAACGTGAACTTTGCCGAGAATCCGATCGCGCTGTCGGTTGTGCTGAGTATATTTGGCGTCTACCTCATCCTCGCCATCTACTCGCGCCGGGAGGACAAGAAGGATATCGAAAGGGCTGGTGTTACTCCACTGGAAGATAACGACCCGTCTGACCGCTATCACTATGAAATCACAGTCTACACAGGTTTCGGCAAAAAGGCCGCCACTACTGCACAAGTCTCGTTCATCCTCGCTGGCGACGAGGGCGAGGGAGAGCCCCGGATTCTCAAAGATCCCAAAAGGAAGACCTTCCAAAGACGGGGCATCGACGTGTTTCTCGTCACGTACCCGGAGAGCCTCGGAGAGATCAACTACCTTCACATCTGGCACGATAACACAGGGAGGTCACCCTCGTGGTACCTAAGCCGAGTAATGGTTGAAGACATCAACAACGACAAAAAGTACATGTTCATCAATGAAAGCTGGCTGGCCGTGGAGGAAGGCGACGGCACTGTGGACCGGCTCATCCCGGTTGCAGGAAAAGACGAAATGACCAGCTTCAACCACCTCTTCTACTCTACAACTCAGAAGAATCTGGCCGACGGTCACTTGTGGTTCTCCATCTTTATGAGGCCCGCGCGCAGTCGCTTTACACGTCTTCAGCGAGTTTCGTGTTGCCTGACCCTACTCTATTGCTCCATGTTGGCAAACGCTATGTTCTATAACATCGGAGGTGAGACAGATCCCTCACAGACTCTTCAGATTGGACCGCTTGCTTTCAGCCCAGCGCAAGTGGGCATTGGTATCATGAGTAGCTTGGTAATCGTCCCTGTCAACATCTTTCTCGTCGCTGTGTTCAGGGGTGTCGAGCCGATGCCGACCCCGGCTGAGTTGAAGGAGAGGAAGAGCCGTAAGTATTGGTGGTTCTACGAGATCTTTTTCTGCTTCTTCGACCGCAACCCGAAAAAGAACGACTTCATTCAGATTCTGCACAAGAACCACAAACCCGATGATTTCCTAGACCTCAGCTCTTCGAGTCGCACAAATCTCGCTTTCAACGACAGCTTGGATCTTGGCCTTGGTGACGATGACATCAACTTTAGGATTTCCAAACAAGAGAAACGAGAGGAGATGGAGAAGAAACAGAAGAAGAAAAAGAAGAAGAAGAAGCAACTGCCGTACTGGTTTCTGTACATCGCGTGGGTCGTGTGTGGTCTGACTTGCTTCACCTGCTCCTTCTTCGTGGTGCTCTACGGTCTCCAGTTCGGTCATGACAAGTCTGCTCAATGGATCTCCTCCATGTTAGTGTCGTTCTTCCAAGACGTACTGGTCAGTCAGCCGATCAAGGTGGTCGCCATCGCGCTGATCATCGCCGCAATCATCAAGAAGCCGCCAGAGGAGGAGGATGATGGCGACAAGAAGAAGCTGGAAGATGAGGACTGGATGCACGATGACGGAAACTCCGAGAAACGAGACAAGAGAATGAGACCAAAGGGACTTATTCGCCTGAAACCGCCCAACAAAGAAAAGCTAGAGAAAGATCGTCAGCAAAGGTTCAAGGAGATGAAGATGAGTGCCATGATCAAGGAGGTCACACTCTACACGTTCTTTGTGGCCTGCCTTTGCATTGTCAGCTACTCTCACAGGGATCCCACCTCCTTCCAGTTCAGGCAGTCAATGTACAACACGTTTGTTTCTGGAACTTACGGCGGAGTGCGTTCTTTCGATTCGATTGGAAGCCGCGAGAATTTCTACGACTGGGCGAAGACCACACTCATGACCAGCTTGTTCAAGAACACGTGGTACAATGGCAATCCGTACGACGTGGGCTTCACTGGGGATGGCATCGCTTACGTGGTTGGCGGGGCGAGGATGAGGCAGTTGCGCGTAGAGAAACATTCCTGCGAGGTTCCGTATCAGTTCAACAAGCTTGTCCATAACTGTAAGACTTGGTATGGCTTCTTTGCCGAGGATACAGGCCAGTATGACATAGCCTGGGAGCCACTGAAAAATGAGTCGCTCTACAAGCCGCCATTTACCTTCAAATCATGGGAGTTTTACGAATCGGCGGAATTGGACTCTATGCCGTTTATGGCATACGTTTCAAGCTATGGCGGCGGTGGGTACGCGGCCGAGCTGGGTCAGACAGAGGAACACGCGTTACGAGTCATCAAAACACTCGAGAATAACACTTGGATCGACTCGCAAACGCGCGCGGTCTTCACAGAAGTGTCCACGTACAACCCAGTGTCTAACCTTTTCTGTGCCATGACTTTCGTGGTAGAGTTCCTTCCCACAAATGGTGTCTATCTGTACATGGATCTCAAAGTGTCCAGGCTGTTCGCTACCGGGGGTGGCTTTGAAACGTTTCTCGTCGTTTGTGAGTTTCTTGTGGTTGTATTCTTCCTGATTTTCATTTACCAGGAACTCAAGCAGCTCTACCGAATGAGGAAGGCATACTTTAAGGATTTCTGGAACAACATCGAATTCACCATGGTTATTCTCGTGCTTGCCAGCGTGTGCATGTTCCTGATGAGGCTGAAACTCGTGGAAAGCGCACTGACCAAACTTGAGAAGCAGGGTAACACGTTCGTGAGCTTCAGCCGAGTGTCGTCTTGGAGCGAAGCCTTCATGATTGTAGTTGCCTTATTGGTTTTCACGACATGGCTCAAAGGAATCAAACTTCTTCGGTTTAATCCGAGGATCCTGATGTTGACCAGGACCCTGAAAGGCGCGGCAGGGCCTCTGGCAACCTTCTCTGTGGTCTTCCTTGTCTTCTTCATGTCCTACGCGTTGTTTGCTTTTGCTGTCTTCGGTAAGGACATCCAGTCGTTTTACAATTTTGTCACGACTGCTGAATCCGTGATGGGACTTCTTTTGGGATCCTTTGATTATGGAGAGATAGAGGAAGCGCAGCCGATCTTGGGTCCGATTTTCTTTTTCACTTTTATGGTATTTGGTAACTTTATTATCATGAACATGTTCTTGACGATTATCATGGACGTATTTGCCGAGGTGAAGGAACAGCTGTCCGAGCAAAACGACAGCGAGTTCGAGGTGGTCGAATTCATGGTTCGGAGATTCCGAAAGTTCACCGGAATGCAGCCGAACAAAGTGAACATGGAGGATGCGGAGGACAAAAAAGAAATGGAGGAAAGACTCAAGGATGACATGACGGTGTTTAAGGTGAAGAAGAAGAAGAACCGCCACCGCAAGTTGCAGCCCATGGACCTCGTGGCGCAGAGGTTTTCGCGGTTGGACGACTCCCTCAAGGGATTCTGTTGCGATGAGTGGGCAGAGGAGCGCATGCTAGATGATATCGTGGAGCGCAAATGGGGTATTAACACCGACGAGGTTAACAGATCCGCTCAGTGTGAGCTGAAGCTCGCTGAGCAGCAAGAGGCGTTTCGGTTGGACATGTACGCGGCGCTTGACAACTACGAGGCTTCCCCCACGGATGAGGACGCCTTCACCTTCAGTTTCCCGGATGGAGAATTCAAACGAGACTTGAGCGAGGCTTAAACCTTTACAATATGCATGCGGGATGTTCCACCAGTTCAGAAGCAAGGCATAGCCAGTCTGTACATAATGCATGCCGGATATCCTATCAGTCCACCTCGAAAGCAAGGCTTAAACGTGTCCATAATGCATGCATTATATTCGGCCATTTCAGCCTTTTCCTATTGATACTACACGTTATACTTAGCTGGCTCTTGAGTCTGGAGAGAGGGTCTCGGAATTTACCATGAATTTACTTGTATCCTGCTTTAAACGAGTCCGATTCTATTGAAACTTAAAAAACCCTGTATTTCAATCAAGTAATTGTTACCTATTTATGTTTTGACAAGGGCTAAACCAATTAAGCTTGCTTGAATTTTGCATTATTTAAGTCATTTTAAGCCTTACTGTTTTCTTTTATATTTTTGCAATCATTTGCCGTTTTATTTGTGTTCATTTGAGTCTTTATCTTAAAACTGTACTGAAATCTTGTATTGAACTTATATTATAGCAATGTTACTAATATATCCTAAATACACTTAACAGAGCCTCGATGCAATATTGGATTGGATTGGATTTTTTAAACAGCATAGCAGACTTTACTTTAAAAACTAATTTATCTCTAAAATAGTTTTGTATTTTATTGCAGAACCATAATTCGAACGCATTTCGTGGTAAAATGGGTTTTCTTTGTCATAAAACCAACATCCACCGCTTGGAAGAAGTCTCAGTTTGCGGGGCTGGGTGAAAGGAACTTTTTTAATAGCTTTTGGTCGTTGTTTTGCGCATAATTATGAATAAGAATTTGAAAATGATAGTACTGGC
