# Supplementary material for: Cnidarian hair cell development illuminates an ancient role for the class IV POU transcription factor in defining mechanoreceptor identity
Source: eLife. 2021 Dec 23;10:e74336. doi: 10.7554/eLife.74336 (PMC8846589; doi:10.7554/eLife.74336)
Supplement: Figure 8—figure supplement 1—source data 2. [file elife-74336-fig8-figsupp1-data2.docx]

MTTKRYLCVLLCLTSYLTYSSGMMTQVRKEEYLGCFTESEESRVFSSGPGDYDPHDISPIRCLEQCGIKYKYAALQDGRLCLCSNTLPGTPKLDDSECNTPCPGSSKWPPSEHYLKCGG (WSC carbohydrate binding domain)PLKNSVYNAGERILGFTLQKIESLNILEPVNIHGGITN GINVSYVFDLGDGTLVTKPSSEPKARHIYDKPGSYVVTATASNIISGEVVASEVYNVDDPRNNIRLTCPRAAEVGQIVECNGTMDRGSRVNSTFVFSDGRTDRMSISSRYYSAGTIVPRGNDSSVIPVLNTPGTILIPAYEFQHDGQVTHWDFEIVEKGTIKLMILRPECSAGEEYCTSTRSCKISSSSCLPLKQKKCSSDEMFCMIQKRCVSNAYTTTQDANNNPVKVYTSSSTCPIQAPYQWSEPRADYRILFVQEISLETIGHHISAIPLAQQPFVKEGDILGWLPVTGYLAYKSVADHEGASFEYSSGVSAVNDKLLRSGSTTLHQKHFVFAAHYAHVAKFVVRNRFGTPGLKSLTSNITEPLYLYIDYPIRNVTFEASKFANTNDSVEFLVPEHPGTNTTYFWDFGNGESLYTHLPSISYAFPTEGVFYVSLRAENSISHTVLTFPISIFDPILEFEYKSPIKANALGTETLIEWKTSRGTNITFVVDFGDATPRYSAVTTLSGGRAVDTRHTYSAVGNYTVTVYAFNRVGPNITIVSYAVVEVPLEGLEFSVPNPHITKNIYLAAGDTMTVSRHYQKGTNIKCSCDFRDGTPPVLTTSQDMSHTYTNAGTYHVEITCFNDVNSITKPLNGTVVVQELQAITGLTVLTSATKFGTRSELLLEMATGSVFVCEWDFGDGNKTSTDFSFMGQTMYYTYVAVDTYNVAVTCTNRVGSVTARAVAPVDIPIDGVIISNNKRYIKVGEPVRLDVTVQKGTRMLYTVSYGDASTGSLSRDAAKAPSLADHESFTHAYATDGSYTVKVNVSNSYGWKEETLGETIMAQYPVEGIILRSNSPVRLSSGNVTYFISVLEGANPPTGAYAVWSFGDNSPVTTPEPIYDLRQKTYMRSHRFMINNTFTTTVNISNQVSHEVLAIDVRIQMLVGVIITPLLVTNATLFTITNGYGPEMNYFEVNKLIAFTSSSQLGDRTWAWEFGDGASTNVSSIPTSTHTFNTSGTYAVRVVVNNFLDVLEAEKTVFIXDPVGNVTLSSQLPTYYREPTVFNFQVTSRGSQSCLKLSLGDNNGAIFGQRHCRPSVMVANVTFIPVPENQTSFNYSYMYIYRGNYSVELTLWNFVSSQSVVWPIEIADLP(PKD domain)CDYPIVRIDSEGTKTSPRKVKKSEPLVLPADVRYKCPVGKRIIFSWKAYEVTLLNPDDESRPFNLPVNEIKTFDLPARDTIMDAGSIKIKERTFPFITLKFTLEVGFVGSDRDLTHFTHSHSVWIEVEKSLLYAVIRGGQRKSVGYEMDMLLDGSESKDPDNPTNTTGIVYTWWCRRDEESFPSAFDAPNPTGGCYGNGNYQLNGSTSEISVYTGAFLQNAVYVFRIKVVKEEREALFDQYITILPGQPPTMNLKCNFNCLAKTNPIERLVMETTCQDCKPTDILGYEWSLHRLLLGKDPDQIDSWETINPTSWAVNTSTGIDKGNLVINSHFLEPSRSYFLRLNAWKPGGYPGGFVEHRFTVNTAPTSGSCSVDPLEGFALDTTFQVKCDGWVDPDTPLKYLVELRNGADIVPISDGFEPYTSAVFPLGKEENNYTLTVNVKVMDMFFLDATTKFSVRVTEPITIDYNEVGGSVASAAGSGNAQEATQVTNA(REJ module)VCSVLNAKACKEEDDPNAKDARADFRGEV AKSMATLPVDSFDGAAQKGEALNGLTAMPDEIKEDAQEAVTDAMNEIGDFLAKDNSGRNLDNTAKSLISGIGNIVGASSNTAKKALNSTCGDPSKSTNNTKKALDLVEIVSSACMKQLVAGDKPKXIKTDNIDLAIGRKDLSDLANDDEDESEGDTGGFSLPDPAMLFGGANASTEEGATSGIGSTMTAMGDNPFPGGSDDLNSKTIGLSLTDGNGNPLDLAGQTLEMYVPRDLKKNPLKPMELNHFGPNDPVMRVHKFNRTTNLTAIAVEIQPFDPQIKFRIHVRFETRPSATHFHWNHTFPSLEEAAKMKRRPHPFTFVINHVVLRDTLLSSNASDNGTVFNSTMGSYFLGIKAINKDSLSSANTSYAMRIYLPACKSFDVDTNTWTTNGCVVGNKTRANITHCVCR(GPCR proteolytic site)PGEDEPEDIDPTAVPPGAAIGNAASSTGDDTSSGGPVRVRRFKRKKVFK LSLASSFFPAPNPIDFDKVFANVNFAENPIALSVVLSIFGVYLILAIY(TM1)SRREDKKDIERAGVTPLEDNDPSDRYHYEITVYTGFGKKAATTAQVSFILAGDEGEGEPRILKDPKRKTFQRRGIDVFLVTYPESLGEINYLHIWHDNTGRSPSWYLSRVMVEDINNDKKYMFINESWLAVEEGDGTVDRLIPVA(PLAT domain)GKDEMTSFNHLFYSTTQKNLADGHLWFSIFMRP ARSRFTRLQRVSCCLTLLYCSMLANAMF(TM2)YNIGGETDPSQTLQIGPLAFSPAQVGIGIMSSLVIVPVNIFLVAVF(TM3)RGVEPMPTPAELKERKSRKYWWFYEIFFCFFDRNPKKNDFIQILHKNHKPDDFLDLSSSSRTNLAFNDSLDLGLGDDDINFRISKQEKREEMEKKQKKKKKKKKQLPYWFLYIAWVVCGLTCFTCSFFVVLYG(TM4)LQFGHDKSAQWISSMLVSFFQDVLVSQPIKVVAIALIIAA(TM5)IIKKPPEEEDDGDKKKLEDEDWMHDDGNSEKRDKRMRPKGLIRLKPPNKEKLEKDRQQRFKEMKMSAMIKEVTLYTFFVACLCIVS(TM6)YSHRDPTSFQFRQSMYNTFVSGTYGGVRSFDSIGSRENFYDWAKTTLMTSLFKNTWYNGNPYDVGFTGDGIAYVVGGARMRQLRVEKHSCEVPYQFNKLVHNCKTWYGFFAEDTGQYDIAWEPLKNESLYKPPFTFKSWEFYESAELDSMPFMAYVSSYGGGGYAAELGQTEEHALRVIKTLENNTWIDSQTRAVFTEVSTYNPVSNLFCAMTFVVEFLPTNGVYLYMDLKVSRL(TOP domain)FATGGGFETFLVVCEFLVVVFFLIFIYQELK(TM7)QLYRMRKAYFKDFW NNIEFTMVILVLASVCMFLMRLKL(TM8)VESALTKLEKQGNTFVSFSRVSSWSEAFMIVVALLVFTTWLKGIKLL(TM9)RFNPRILMLTRTLKGAAGPLATFSVVFLVFFMSYALFAFAV(TM10)FGKDIQSFYNFVTTAESVMGLLLGSFDYGEIEEAQPILGPIFFFTFMVFGNFIIMNMFLTIIMDVF(TM11)AEVKEQLSEQNDSEFEVVEFMVRRFRKFTGMQPNKVNMEDAEDKKEMEERLKDDMTVFKVKKKKNRHRKLQPMDLVAQRFSRLDDSLKGFCCDEWAEERMLDDIVERKWGINTDEVNRSAQCELKLAEQQEAFRLDMYAALDNYEASPTDEDAFTFSFPDGEFKRDLSEA
