# Supplementary material for: Cnidarian hair cell development illuminates an ancient role for the class IV POU transcription factor in defining mechanoreceptor identity
Source: eLife. 2021 Dec 23;10:e74336. doi: 10.7554/eLife.74336 (PMC8846589; doi:10.7554/eLife.74336)
Supplement: Figure 8—figure supplement 2—source data 1. [file elife-74336-fig8-figsupp2-data1.docx]

>Drosophila_melanogaster_PKD2_Q9VK95

WTVLAFYRTGGYTVNL--DYDKDRNVKIINDLKDIHWLDRGSRLCLVEFNLFNENTDIFQSIKLIAEIPPTGGVIPQAHLQTVKMYSFFT--DRSMLMTVIYIFWYIMVIYYTIYEITEIRKSGIKIYFCSMLNILDCAILLGCYLALVYNIWHSFKVMSLTARAH-SDV-TYQSLDVLCFWNIIYVDMMAILAFLVWIKIFKFISFNKTLVQFTTTLKRCSKDLAGFSLMFGIVFLAYAQLGLLLFGTKHPDFRNFITSILTMIRMILGDFQYNLIEQANRVLGPIYFLTYILLVFFILLNMFLAIIMETYNTVKGEITQGR

>Homo_sapiens_PKD2_AAC50520 A new protein sequence entered manually

WGIIATYSGAGYYLDL--SRTREETAAQVASLKKNVWLDRGTRATFIDFSVYNANINLFCVVRLLVEFPATGGVIPSWQFQPLKLIRYVT--TFDFFLAACEIIFCFFIFYYVVEEILEIRIHKL-HYFRSFWNCLDVVIVVLSVVAIGINIYRTSNVEVLLQFLE-DQN-TFPNFEHLAYWQIQFNNIAAVTVFFVWIKLFKFINFNRTMSQLSTTMSRCAKDLFGFAIMFFIIFLAYAQLAYLVFGTQVDDFSTFQECIFTQFRIILGDINFAEIEEANRVLGPIYFTTFVFFMFFILLNMFLAIINDTYSEVKSDLAQQK

>Mus_musculus_PKD2_O35245

WGIIASYSGAGYYLDL--SRTREETAAQLAGLRRNFWLDRGTRAAFIDFSVYNANINLFCVVRLLAEFPATGGVVPSWQFQPVKLIRYVT--AFDFFLAACEIIFCFFIIYYVVEEILEIRIHRL-SYFRSFWNCLDVVIVVLSVVAMVINIYRMSNAEGLLQFLE-DQN-SFPNFEHVAYWQIQFNNISAVMVFLVWIKLFKFINFNRTMSQLSTTMSRCAKDLFGFTIMFSIIFLAYAQLAYLVFGTQVDDFSTFQECIFTQFRIILGDINFAEIEEANRVLGPLYFTTFVFFMFFILLNMFLAIINDSYSEVKSDLAQQK

>Capitella_teleta_ELT90189 A new protein sequence entered manually

WGYITTYGGGGFYQDL--ALNKADSLALIADLKQNLWLDRGTRVVFVDFTVYNANINLFCVIRLVVEFPATGGAIPSWNFRTVKLIRYVS--ASDYFVMACECIFVLYVIYYIIEETLEIKRHKL-GYFKSFWNILDIIVLMIAVCCIGFNVYRTVAVADMLDTLLDSPN-EYADFEFLSYWQVVFNSALAIMVFFAWIKVFKYISFNKTMTQLSSTLARCAKDLAGFAVMFFIIFLAFAQLGYLIFGTQVKDFSSFDDAVFTLFRIILGDFNFHELEQANRVLGPAYFILYVFFVFFVLLNMFLAIINDTYSEVKEEISNQK

>Nematostella_vectensis_PKD2-like_XP_032219880 A new protein sequence entered manually

WGRMTTYSGGGFTQLL--APTKAETQQIIEDLKQNLWLDRGTRAVFIDFTVYNANINLFCIVRLVFEYPPTGGCIPSFNFRTVKLIRYVD--TFDHFVMACEGIFILFIIYYTIEEGLEIKKHKM-KYFKSFWNVLDVVVIMLGYVAVVFNLYRTMAVGDLLSSLLEDGS-QYANFDSLGFWQTQFNNMVAVAVFFAWIKVFKYISFNKTMTQLSSTLSKCAKDVAGFAIMFFIIFFAYAQLGYLIFGTQVRDFSTFEDSIFTLFRIILGDFDFHQIENANRVLGPLFFMTYVFFVFFVLLNMFLAIINDTYAEVKSDIASQE

>Acropora_millepora_PKD2-like_XP_029208330 A new protein sequence entered manually

WGRITSYSGGGFTLLL--EATKAKTEALIDKLKKNLWLDRGSRAVFIDFTVYNANINLFCIVKLLFEYPATGGCIPSFNFRTVKLIRYVN--TMDHFVMACEGIFILFIIYYTIEEILEIKKHRL-KYFKSFWNVLDIIVIFLGYVAIVFNLYRTVTVSDLLKGLLANNK-QYANFDSLGFWQTQFNNMVAIAVFFAWIKVFKYISFNKTMTQLSATLNNCAKDVGGFAVMFFIIFLAYAQLGYLVFGTQVRDFSTFPDAIFTLFRIILGDFDFHALEAANRVLGPIFFITYVFFVFFVLLNMFLAIINDTYAEVKSNIASQK

>Caenorhabditis_elegans_PKD2_Q9U1S7

VGTIASYGGGGFVQRL-PVAGSTEAQSAIATLKANRWIDRGSRAIIVDFALYNANINLFCVVKLLFELPASGGVITTPKLMTYDLLTYQT--SGGTRMMIFEGIFCGFILYFIFEELFAIGRHRL-HYLTQFWNLVDVVLLGFSVATIILSVNRTKTGVNRVNSVIENGL-TNAPFDDVTSSENSYLNIKACVVFVAWVKVFKFISVNKTMSQLSSTLTRSAKDIGGFAVMFAVFFFAFAQFGYLCFGTQIADYSNLYNSAFALLRLILGDFNFSALESCNRFFGPAFFIAYVFFVSFILLNMFLAIINDSYVEVKAELARKK

>Human_PKD1_AAC37576 A new protein sequence entered manually

WGSCAVYDSGGYVQEL--GLSLEESRDRLRFLQLHNWLDNRSRAVFLELTRYSPAVGLHAAVTLRLEFPAAGRALAALSVRPFALRRLSA--GLSLPLLTSVCLLLFAVHFAVAEARTWHREGRW-RVLRLGAWARWLLVALTAATALVRLAQLGAADRQWTRFVRGRPR-RFTSFDQVAQLSSAARGLAASLLFLLLVKAAQQLRFVRQWSVFGKTLCRALPELLGVTLGLVVLGVAYAQLAILLVSSCVD---SLWSVAQALLVLCPGTGLSTLCPAESWHLSPLLCVGLWALRLWGALRLGAVILRWRYHALRGELYRPA

>Mus_musculus_PKD1_O08852

WGYCAVYDSGGYIQEL--GLSLEESRARLGFLQLHNWLDSRSRAVFVELTRYSPAVGLHAAVTLRLEFPVAGHALAAFSVRPFALRRLST--GLSLPLLTSVCLLLFALYFSMAEVQTWRKDGCA-CTARPDTWARCLLVILTAATGLVRLAQLGIADRQWTHFVQDHPR-HFTSFDQVAQLGSVARGLAASLLFLLLVKAAQQLRFVRQWSVFGKTLCRALPELMGATLGLVLLGVAYAQMAILLISSGAD---TLYNMARAFLVLCPGARVPTLCPSESWYLSPLLCVGLWALRVWGALRLGAILLRWRYHALRGELYRPA

>Mytilus_coruscus_PKD1L2_CAC5406047 A new protein sequence entered manually

VGEISTYKGGGYVAMF--ERNVERTNELIAHLRKDVWLDVYTRGVFLEFTVYNPNLNLFGSMIMLVEFMQSGGAVTRMEFKIFRLLSYIG--GMGVVVILFEVLYACFTLYFFVHFVKKLKKERK-KYFNSFWNKLEFALMLFCVTVIAMYALKHILTSVAMNALKDRERADNVNFQSLAAYDELYSYMVGIVVFLATIQFLKLLQFNKKMNMLGDTVNLASKDLKVFSFAFIMYFLAFTIFGFLLFGSTLYAFAGFISAAESMFAFTLGSFDFAAMEASQKILGPIFFFLFIFIVYVGLMSIFLTIIADAFATVKEDVANQT

>Acropora_millepora_PKD1-like__XP_029194885 A new protein sequence entered manually

MGHLALYRGGGYNTDL--SSSAEEAYSVINYLKSHQWVDKYTRAVFVEFTVYNVHSNLYCVANLLLEFTAAGGVLPFIQLLSTRIDRYVG--NFLIFVLICEVTFVLFSLYFTYREFKRFIKMGLKQYLAEFWSWIELTQIGLSWTCVVLYGIRFALDRHTKTNFRNNPQ-KFVDFHHLALVDMLFGYVYAFVVFLTSVKFLRLFRFNRRMSLLGSTISASARELFHFGIIFGLVFVGFSHLCYLVFSRELYKFHTFLTTIETLISVMLGKFSYVSLERTNRVLGPIMFFFYSIGVVFILVNMFLSIIIENFKRVKNDNDLQS

>Trichoplax_sp._PKD1-like__RDD45301 A new protein sequence entered manually

WGQYHIYYGGGYVVEL--SRNESQSQLTLNQLQQQGWIDHQTRAILIEFTIFNAQVNLFSVVTLLAEFPATGGIMPFVEIQTIRLFRDHY--SAGMLVIVCEIIFVSFIAYYIYRELSKLKRLSL-RYFSGFWNIIEFTIISLAVAAVSIYFFRLQVTRNALAQCRSQSR-HFINFHYVATWDNLYIHLFAFLIFFGTIKLIRLLRFNRTIAILSITLRSAAKEILMFLVVFTVIFLTFAQFAYLIYGRTINSYNTFIKTLESQFNMMIGRFYAKIMYSSLRDVSPLYFFCYTFLVSWVLINMFLTLIIKSFEKVKSCCNQIN

>Hydra_vulgaris_PKD1-like_2_XP_012558373 A new protein sequence entered manually

IGYLATYTGGGYVADL--GNYPLKSHQVINDLHQNKWIDGRTRALFLEFSTYNPQVNLFGIVNLLLEFSPSSAVEFFSSIHIARLYTFAG--ETATLTLVCQIFVVFFFLIAMYKEAKKIYKMKK-LYFRGFWNLYEFFLIILLLITTGVFFSRVMLVKKAVKSIQEDQK-KFVSFNRIVQWDQLFSGLTSVLVLLTCIKSIRILQYNKTISLFVLTLKKSASPLAAFFLIFAIFFTSFTAWAYLMFIPYLPEYSNYISASESVMSLLLGSFKFKDIVSAKPVLGSLWFTLIMIFGVMYIMNVFLTIVMETYASVNKDLSMTS

>Nematostella_vectensis_PKD1

MAYVSSYGGGGYAAEL--GQTEEHALRVIKTLENNTWIDSQTRAVFTEVSTYNPVSNLFCAMTFVVEFLPTNGVYLYMDLKVSRLFATGG--GFETFLVVCEFLVVVFFLIFIYQELKQLYRMRK-AYFKDFWNNIEFTMVILVLASVCMFLMRLKLVESALTKLEKQGN-TFVSFSRVSSWSEAFMIVVALLVFTTWLKGIKLLRFNPRILMLTRTLKGAAGPLATFSVVFLVFFMSYALFAFAVFGKDIQSFYNFVTTAESVMGLLLGSFDYGEIEEAQPILGPIFFFTFMVFGNFIIMNMFLTIIMDVFAEVKEQLSEQN

>Strongylocentrotus_purpuratus_Receptor_for_egg_jelly_3_(PKD1)_Q95V80

WGHATSLPSSGYIWVL--GSMYEEAKDSLAEMVDARWLDARTRALFVEWTSYNANTNLFCVVTFLMETPASGGLLKLPEVQAVRLHRYAA--NYKLFVILCEILFVVALFFVMYREYVRYKPIGIRKYLSDKWNLLEIAIIVNCIVSAGLYIYRYVITKQLFKQMRDESV-RFVGFRTAATSDNALGYSLAVIIILSCVKFLYLLRLNPRMYLLTSVISDCYHEVIAFTFLIFILILSFAFPMTIMFGSNLPDYRDITKTALNLFITLPDNFVYEDLKSVQRVLGPLILLLFQFLSCYLFLDLLIAALNESMVTIRRHPPPPS

>Mus_musculus_PKDRE_Q9Z0T6

YGVLNTYGSGGYVFYFFPGQQMFNSTVRLKELEGKNWLDELTWAVIVELTTLNPDTSLMCSISVVFEVSPLGVVNSSLSVYSFSLADFNRKTSSEIYLYAAI---LIFFCAYVVDEGYIIRQERA-SYIRSVYNLLNFSLKCMFALLIVLFFWKYFLATKMVQLYLADPE-AFIPFHAVSRVDHFMRIILAFLLFLTILKTLRYSRFFYNVRLAQKAIQAALPGICHTALVVSIYSFMYVAFGYLVFGQHEWNYSNMIHATQTIFSYCVSAFQNTEF-SGNKVLGVLFLSSFMLVMICIFINLFQAVILSAYDEMKQPVYEEP

>Homo_sapiens_PKDRE_Q9NTG1

YGLLHTYGSGGYALYFFPEQQRFNSTLRLKELQESNWLDEKTWAVVLELTTFNPDINLFCSISVIFEVSQLGVVNTSISLHSFSLADFDRKASAEIYLYVAI---LIFFLAYVVDEGCIIMQERA-SYVRSVYNLLNFALKCIFTVLIVLFLRKHFLATGIIRFYLSNPE-DFIPFHAVSQVDHIMRIILGFLLFLTILKTLRYSRFFYDVRLAQRAIQAALPGICHMAFVVSVYFFVYMAFGYLVFGQHEWNYSNLIHSTQTVFSYCVSAFQNTEF-SNNRILGVLFLSSFMLVMICVLINLFQAVILSAYEEMKQPVYEEP
